# The Role of Rosmarinic Acid on the Bioproduction of Gold Nanoparticles as Part of a Photothermal Approach for Breast Cancer Treatment

**DOI:** 10.3390/biom12010071

**Published:** 2022-01-04

**Authors:** Tânia Ferreira-Gonçalves, Maria Manuela Gaspar, João M. P. Coelho, Vanda Marques, Ana S. Viana, Lia Ascensão, Lina Carvalho, Cecília M. P. Rodrigues, Hugo Alexandre Ferreira, David Ferreira, Catarina Pinto Reis

**Affiliations:** 1 Research Institute for Medicines (iMed.ULisboa), Faculty of Pharmacy, Universidade de Lisboa, Av. Professor Gama Pinto, 1649-003 Lisboa, Portugal; taniag1@edu.ulisboa.pt (T.F.-G.); mgaspar@ff.ulisboa.pt (M.M.G.); vismsmarques@ff.ulisboa.pt (V.M.); cmprodrigues@ff.ulisboa.pt (C.M.P.R.); 2 Instituto de Biofísica e Engenharia Biomédica, Faculdade de Ciências, Universidade de Lisboa, Campo Grande, 1749-016 Lisboa, Portugal; jmcoelho@fc.ul.pt (J.M.P.C.); hugoferreira@campus.ul.pt (H.A.F.); 3 Centro de Química Estrutural, Faculdade de Ciências, Universidade de Lisboa, Campo Grande, 1749-016 Lisboa, Portugal; apsemedo@fc.ul.pt; 4 Centro de Estudos do Ambiente e do Mar (CESAM), Faculdade de Ciências, Universidade de Lisboa, Campo Grande, 1749-016 Lisboa, Portugal; lmpsousa@fc.ul.pt; 5 Central Testing Laboratory, University of Aveiro, Campus Universitário de Santiago, 3810-193 Aveiro, Portugal; linamcarvalho@ua.pt; 6 Comprehensive Health Research Centre (CHRC), Departamento de Desporto e Saúde, Escola de Saúde e Desenvolvimento Humano, Universidade de Évora, Largo dos Colegiais, 7004-516 Évora, Portugal; david.ferreira@uevora.pt

**Keywords:** rosmarinic acid, gold nanoparticles, photothermal therapy, breast cancer treatment

## Abstract

Breast cancer is a high-burden malignancy for society, whose impact boosts a continuous search for novel diagnostic and therapeutic tools. Among the recent therapeutic approaches, photothermal therapy (PTT), which causes tumor cell death by hyperthermia after being irradiated with a light source, represents a high-potential strategy. Furthermore, the effectiveness of PTT can be improved by combining near infrared (NIR) irradiation with gold nanoparticles (AuNPs) as photothermal enhancers. Herein, an alternative synthetic method using rosmarinic acid (RA) for synthesizing AuNPs is reported. The RA concentration was varied and its impact on the AuNPs physicochemical and optical features was assessed. Results showed that RA concentration plays an active role on AuNPs features, allowing the optimization of mean size and maximum absorbance peak. Moreover, the synthetic method explored here allowed us to obtain negatively charged AuNPs with sizes favoring the local particle accumulation at tumor site and maximum absorbance peaks within the NIR region. In addition, AuNPs were safe both in vitro and in vivo. In conclusion, the synthesized AuNPs present favorable properties to be applied as part of a PTT system combining AuNPs with a NIR laser for the treatment of breast cancer.

## 1. Introduction

Breast cancer is a major worldwide health problem affecting millions of people and being accountable for thousands of deaths. According to GLOBOCAN estimates for 2020, there were about 2.3 million new cases of breast cancer and 6.8 thousand deaths [1]. Limiting the geographic area to the United States of America and according to the American Cancer Society data from 2021, one in eight women will be diagnosed with breast cancer over the year of 2021, and about 43,600 women and 530 men will die from breast cancer. This means that it is expected that every 13 min one woman will die from breast cancer in 2021 [2]. Furthermore, attending the GLOBOCAN 2020 data, it is predicted that there will be an increase in both breast cancer incidence and mortality over the next years, being estimated to reach 3.0 million new cases and 1.0 million deaths by 2040 [1]. In response to the constantly increasing cases of breast cancer, a huge effort has been made to encourage more frequent and easily accessible breast cancer screenings, as well as to find new and more effective diagnostic and treatment strategies [3,4]. Several treatments have been proposed for breast cancer [5,6,7], namely surgery [8,9], radiation [10] and/or chemotherapy [11], immunotherapy [7], hormone therapy [5,11] and targeted therapy [12]. Nevertheless, the outcomes of the currently available strategies are still typically associated with high impact both on patient’s life and self-esteem [13], and/or lack of specificity towards cancerous cells, which increases the chance of suffering from extensive adverse side effects [14]. Moreover, breast tumors are also associated with treatment resistance [15,16]. Considering the aforementioned issues, there is an urge to establish new, non- or minimally invasive targeted therapeutic approaches to fight breast cancer.

Among the novel therapies proposed for breast cancer, photothermal therapy (PTT) appears as a minimally invasive therapy with likely shorter recovery periods [17,18]. PTT is based on the induction of cancer cell local hyperthermia upon irradiation with a light source, which will then result in cell death that is, to a certain extent, tumor-selective, as tumor cells have limited ability to dissipate heat when compared to healthy cells [18,19,20,21,22]. PTT efficacy depends on one hand on the heat generated locally and consequent possibility of inducing cancer cell hyperthermia (temperature ranging between 41–47 °C [21,23]), and on the other hand, on the ability of the light to reach the target tissues. The light interaction with biological tissues (e.g., scattering, absorption and transmission) is greatly influenced by the light wavelength [24], which in turn determines its ability to penetrate tissues in depth. Among the light spectrum, near infrared (NIR) radiation, with wavelengths between 650 and 900 nm, is less absorbed by tissues [25,26], making it possible to reach deeper tissues. Considering the above, the use of NIR light represents a promising strategy to improve PTT efficacy, even though it only allows reaching tissues a few centimeters deep, thus keeping its potential limited to superficial tumors [27]. The local heating effect of PTT is another aspect drawing attention, not only to reduce undesirable effects of the therapy over healthy tissues, but also to enhance the temperature reached locally at the tumor site. The amount of heat generated is considered a strong limitation for the PTT efficacy in two fronts: on one hand, it must be enough to damage tumor cells without affecting surrounding healthy cells; and on the other hand, it must not be excessively high to avoid resulting in tumor cells’ thermal resistance [28,29]. 

Some of the strategies to improve the local efficacy of PTT rely on the use of photothermal enhancers to simultaneously exhibit active targeting of tumor sites and enhance the heat generated [27,29]. Example of such photothermal enhancers being actively researched are gold nanoparticles (AuNPs) [19,30]. In this regard, AuNPs have been widely used in PTT systems due to their particular ability to convert light energy into heat [31,32,33]. Heat generation by AuNPs occurs in consequence of the surface plasmon resonance (SPR) effect that appears upon AuNPs irradiation with light beams of certain wavelengths and irradiance [33,34,35,36]. In addition to the photothermal properties, AuNPs present other features that have attracted attention for their use in PTT systems, including low toxicity [37] and enhanced selectivity, either via passive targeting through enhanced permeability and retention (EPR) effect of tumors [38,39] or active targeting through the conjugation of specific coatings and/or targeting moieties on their surface [40]. All the aforementioned properties depend, however, on particle’s physicochemical features, namely shape, size, surface charge and functionalization [41]. Several methods have already been proposed for the chemical synthesis of AuNPs [42,43], and thus are considered easy to tune in terms of physicochemical and optical properties. Nevertheless, most of the chemical methods proposed use cytotoxic reagents such as cetyl trimethyl ammonium bromide (CTAB) and citrate [43,44,45], boosting the search for alternative approaches using safer and more environmentally and biologically friendly reagents [46,47,48,49].

The aim of this work was to synthesize AuNPs to be used as part of a PTT system for breast cancer treatment. The PTT approach proposed is based on the combination of local intratumoral injection of AuNPs with NIR laser irradiation to simultaneously enhance the photothermal effect and improve the deepness reached by the light. Thus, as predefined criteria, the nanoparticles must present suitable sizes that enable their retention at the tumor site, minimizing entrance in the blood stream and further accumulation at non-targeted sites. AuNPs properties such as size, shape, surface charge and surface modifications/coatings are known to affect their biological effect [41,50]. It is reported that larger particles (≥200 nm) tend to accumulate preferentially at the injection site [51], and so AuNPs with sizes around 200 nm were of interest. Furthermore, to reach deeper tissues an NIR laser was used, and the AuNPs were prepared to exhibit maximum absorbance at wavelengths within or close to the NIR range (>600 nm). Herein, AuNPs were synthesized using rosmarinic acid (RA) as one of the reducing agents, in substitution of the typically harmful cytotoxic reagents. This experimental approach was adapted from the method previously described by our group [52,53,54,55,56], this being the first report assessing the effect of RA on the resulting AuNP physicochemical and biological properties. Moreover, in previous publications from our group, an aqueous extract of a Lamiaceae species, *Plectranthus saccatus* Benth, was used as the main source of reducing and capping agents [52,53], which has now been replaced by RA. RA is a phenolic compound with well-known biological activities, namely antioxidant, anti-inflammatory, antibacterial and antiviral effects [57]. Furthermore, RA is also known by its anticancer effects [57,58,59], which emphasizes its potential for synthesizing AuNPs aimed to integrate a PTT system for the treatment of localized tumors. The rational for this replacement was based on the fact that RA was identified as one of the main constituents of the extract [52], which in turn is no longer available. In addition, the use of RA aimed to surpass the main drawbacks associated with the use of plant extracts, namely high composition variability depending on plant origin and time of collection [60].

## 2. Materials and Methods

### 2.1. Materials

#### 2.1.1. Reagents

Gold (III) chloride trihydrate solution (HAuCl_4_·3H_2_O), L-ascorbic acid (L-AA), silver nitrate (AgNO_3_), RA, phosphate-buffered saline (pH 7.4, PBS), trypsin, fetal bovine serum (FBS) and dimethyl sulfoxide (DMSO) were purchased from Sigma-Aldrich (St. Louis, MO, USA). Dulbecco’s modified Eagle medium (DMEM) was supplied by Biowest (Nuaillé, France), and penicillin and streptomycin were obtained from Invitrogen (Waltham, MA, USA). *Artemia salina* eggs and artificial sea water salt for *artemia* growth were purchased from JBL GmbH and Co., KG (Neuhofen, Germany). All other reagents and solvents were of analytical purity grade. MilliQ water used in all the experiments was purified through a Millipore system (Millipore, Burlington, MA, USA).

#### 2.1.2. Cell Lines and Cell Culture

In vitro safety and efficacy of AuNPs were assessed in murine healthy fibroblasts L-929 (CCL-1^TM^, ATCC^®^, Manassas, VA, USA), murine breast cancer 4T1 cells (CRL-2539™, ATCC^®^, Manassas, VA, USA) and human breast cancer MCF-7 (HTB-22^TM^, ATCC^®^, Manassas, VA, USA) and MDA-MB-231 cells (HTB-26^TM^, ATCC^®^, Manassas, VA, USA). The MCF-7 cell line represents an estrogen receptor (ER) and progesterone receptor (PR) positive and HER2 negative cancer [61], whereas MDA-MB-231 and 4T1 cell lines represent triple-negative cancer [62,63]. All cell lines were cultured in DMEM with high glucose (4500 mg/L) enriched with 10% of FBS (*v*/*v*), 100 IU/mL of penicillin and 100 µg/mL of streptomycin (henceforward, complete medium). Cells were kept in an incubator (NuAire NU-5500E, NuAire, Plymouth, MN, USA) at 37 °C and 5% CO_2_ atmosphere, and every two days cell medium was changed when a confluence of 80% was reached.

### 2.2. Methods

#### 2.2.1. Preparation of AuNPs

The AuNPs were synthesized through a modified version of a synthetic method previously reported [52,54,55,56], using here RA instead of the plant extract. Moreover, we studied the effect of the concentration of RA on particle’s properties. The RA concentrations tested, and the corresponding nomenclature used to identify the particles obtained are presented in Table 1. Briefly, the AuNPs were prepared based on a mixture of reducing agents (RA, L-AA (2 mM) and AgNO_3_ (1 mM)) with HAuCl_4_·3H_2_O (1 mM) at room temperature (RT), under magnetic stirring (800 rpm) (Heidolph MR3001, Heidolph Instruments, Schwabach, Germany) for 15 min. Afterwards, the AuNPs were stored for 24 h at 2 °C, protected from light. The particles were centrifuged at 1520× *g* for 20 min (HERMLE Z 233 M, Hermle LaborTechnik GmbH, Wehingen, Germany) to remove unreacted reagents, and the pellets were resuspended in MilliQ water. Next, the colloidal AuNPs were stored at 2 °C, protected from light, until being used.

#### 2.2.2. Physicochemical Characterization of the AuNPs

The produced AuNPs were physicochemically characterized in terms of size, polydispersity index (PdI), surface charge, maximum absorbance peak and Au concentration. Size, in the form of hydrodynamic diameter, and PdI were determined by dynamic light scattering, DLS (Zetasizer Nano S, Malvern Instruments, Malvern, UK), using a constant scattering angle of 173° and a temperature of 25 °C. The samples were analyzed in aqueous solution after dilution (in MilliQ, 1:10, *v*/*v*). The data from each sample corresponds to 3 series of 11 measurements each. The surface charge of AuNPs, in the form of zeta-potential, was assessed by electrophoretic mobility (Zetasizer Nano Z, Malvern Instruments, Malvern, UK). The samples were diluted in PBS pH 7.4 (1:10, *v*/*v*) and the analyses were carried out at a constant temperature of 25 °C in 3 series of 10 measurements each. The maximum absorbance peak of the particles was determined by spectrophotometry (Shimadzu UV-160A UV-visible recording spectrophotometer, Shimadzu Europe GmbH, Duisburg, Germany), within a wavelength range between 400 and 1000 nm. The molar concentration of the syntheses in terms of Au content was assessed by Inductively Coupled Plasma Mass Spectrometry (ICP-MS, Thermo XSeries ICP-MS, Thermo Fisher Scientific, Waltham, MA, USA). For the ICP-MS analysis, 100 µL of each sample was digested in a closed perfluoroalkoxy (PFA) tube containing 750 µL of HCl and 250 µL of HNO_3_, by subjecting the tubes to microwave radiation at 190 °C for 10 min. Later, the sample was collected, mixed with 10 mL of ultrapure water, and analyzed by ICP-MS. Based on the ICP-MS results, the recovery yield in terms of Au content or Recovery Yield_Ratio_ (Au) was determined in accordance with Equation (1),

(1) $${\mathrm{Recovery}\;\mathrm{Yield}}_{\mathrm{Ratio}}\;\left(\mathrm{Au}\right)=\frac{C_{\mathrm{Ratio}}}{C_{\mathrm{Au}\;\mathrm{salt}}}\times 100$$

where, C_Ratio_ is the molar concentration of Au present in the AuNPs suspension from that ratio determined by ICP-MS, and C_Au salt_ is the molar concentration of Au present in the HAuCl_4_·3H_2_O solution used in the AuNPs synthesis (also determined by ICP-MS).

#### 2.2.3. Morphological Characterization of the AuNPs

Particle morphology was characterized by transmission electron microscopy (TEM) and atomic force microscopy (AFM). 

For TEM, 10 µL droplets of the AuNPs aqueous suspensions were added to 200-mesh copper grids coated with formvar and carbon and allowed to attach. Then, the excess of samples was removed with filter paper a few minutes later. Subsequently, the material was negatively stained with 1.0% of uranyl acetate for some minutes and left to dry at RT. Observations were made on a JEOL 1200 EX transmission electron microscope (JEOL Ltd., Tokyo, Japan) at 80 kV, and images of diverse grid fields were recorded digitally.

For AFM, 40 µL droplets of the AuNPs aqueous suspensions were added to freshly cleaved mica surfaces and allowed to attach. The samples were left air-drying at RT overnight. Images of the samples were acquired at a scan rate of 1 Hz using peak force tapping and ScanAsyst mode from a Multimode 8 HR coupled to a Nanoscope V Controller (Bruker, Conventry, UK). The tip model used was ScanAsyst-air 0.4 N/m (Bruker, Conventry, UK). The images were prepared using the Image software NanoScope V 1.8.

#### 2.2.4. In Vitro Thermal Activation Studies Using Phantoms

**Figure 1:**
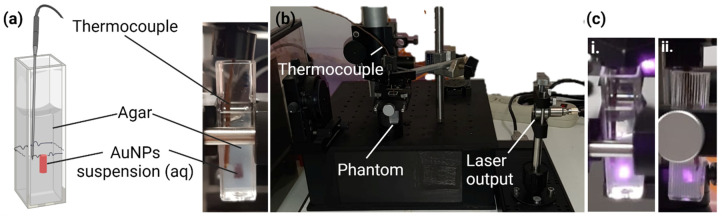
Experimental setup for studying the thermal activation of AuNPs. (**a**) Schematic and real representation of agar phantoms incorporating AuNPs; (**b**) Laser irradiation experimental setup; (**c**) Frontal (**i**) and lateral (**ii**) view of a representative phantom incorporating AuNPs during an irradiation process.

For studying the thermal activity of AuNPs, these were incorporated into agar phantoms, as shown in Figure 1a. Firstly, an agar 1% (*w*/*v*) solution was prepared by dissolution of agar in water under magnetic agitation and heating. Subsequently, 1 mL of agar solution was poured into each polystyrene cuvette and allowed to completely jellify at 2 °C. Then, a small well was made at the center of the agar gel in all cuvettes, where 20 µL of each AuNPs suspension (in equivalent Au concentration in all 4 Ratios) was added in the testing phantoms and a small sphere of black plasticine (diameter of 0.40 ± 0.05 mm) was added for the positive control phantoms. Moreover, the wells of the negative control phantoms were not filled with any solution. Lastly, 1 mL of agar solution was again added to the phantoms to cover the previously filled wells. After having the phantoms ready, they were assembled in the irradiation setup and a thermocouple (Fluke 52 K/J thermometer, Everett, WA, USA) was immersed in the phantom as represented in Figure 1b. As a note, the thermocouple was introduced at the same level of the AuNPs suspensions but in the front left vertices of the cuvette, whereas the particles suspension was placed in the center of the cuvette. Thus, the temperature measured did not represent the maximum temperature reached in the center of the AuNPs, but the temperature of the surroundings instead. For this reason, the temperature of the phantoms was assessed before and after laser irradiation, to later determine the temperature increment caused by the irradiation as an alternative to measure an absolute temperature. A JDSU L4-2495-003 Diode Laser (JDSU, San Jose, CA, USA) coupled to a LaserPak laser diode driver ARO-485-08-05 (Arroyo Instruments, LCC, San Luis Obispo, CA, USA) emitting at a wavelength of 811 nm (in the NIR range) was used. The laser beam was collimated, centered, and aligned to irradiate the cuvette containing the testing samples. The irradiation took 3 min per phantom and an energy density of 9.8 ± 0.5 J/mm^2^ was delivered. The irradiation dose was selected based on previous works [55]. The irradiation time was posteriorly extended up to 10 min to evaluate the evolution of the phantoms’ temperature depending on the time of irradiation.

#### 2.2.5. In Vitro Nanoparticle Internalization Studies Using Light Microscopy

L929, MDA-MD-231, MCF-7 and 4T1 cells were seeded in 96-well plates (200 µL per well) at 5 × 10^4^ cell/mL and allowed to adhere, overnight, in the same culture conditions specified previously. Then, the complete medium was removed, and the cells were incubated for 4 h with the AuNPs in complete medium at a concentration of 128 ± 21 µM of Au. After incubation period, the medium with the potential unbound AuNPs was removed, cells were washed twice with PBS 1×, and new incomplete medium was added. Brightfield optical microscopy images were acquired with Invitrogen EVOS^TM^ FL Auto2 imaging system (Invitrogen, Thermo Fisher Scientific, Waltham, MA, USA).

#### 2.2.6. Safety of AuNPs

##### In Vitro Cellular Viability

L929, MDA-MD-231, MCF-7 and 4T1 cells were seeded in 96-well plates (200 µL per well) at 5 × 10^4^ cell/mL and allowed to adhere overnight in the same culture conditions specified above. The following day, complete medium was removed, and cells were incubated in the same culture conditions for 24 h with the AuNPs in complete medium at a concentration of 128 ± 21 µM of Au. After that period, the medium with the potential unbound AuNPs was removed, and cell viability was assessed by 3-(4,5-dimethylthiazol-2-yl)-2,5-diphenyltetrazolium bromide (MTT) method, a standard technique widely used that evaluates mitochondrial activity [64]. Briefly, cells were washed twice with PBS 1× and 50 µL of MTT in incomplete medium (0.5 mg/mL) were added to each well and incubated at 37 °C in a 5% CO_2_ atmosphere for 3 h. The formazan crystals formed upon reduction of MTT were solubilized by adding 100 µL of DMSO to each well. Absorbance was measured at 570 nm using a BioTek ELx800 absorbance microplate reader (BioTek Instruments, Inc., Winooski, VT, USA). Cell viability in percentage was determined according to Equation (2),

(2) $$\mathrm{Cell}\;\mathrm{viability}=\frac{{\mathrm{OD}}_{t}}{{\mathrm{OD}}_{c}}\times 100$$

where OD_t_ is the optical density of cells incubated with the tested formulations and OD_c_ is the optical density of the control cells, corresponding to 100% of cell viability.

##### Hemolytic Activity Using Human Red Blood Cells

The hemolytic activity of AuNPs was evaluated using ethylenediamine tetraacetic acid (EDTA)-preserved peripheral human blood collected from voluntary donors [65]. The blood was collected in the same day of the experiments. Briefly, the blood was centrifuged at 1000× *g* for 10 min (Beckman GPR Centrifuge, Beckman Coulter, Inc., Brea, CA, USA) to separate the plasma from the erythrocytes. Subsequently, the erythrocytes were diluted in PBS 1× (pH 7.4, USP32) and centrifuged again at 1000× *g* for 10 min. This procedure was repeated three times. Meanwhile, the AuNPs suspended in PBS 1× (pH 7.4, USP32) were distributed in a 96-well plate (100 µL per well, three replicates per condition) in Au concentrations ranging from 256 to 2 µM. Moreover, for positive (100% hemolysis) and negative (0% hemolysis) controls, 100 µL of MilliQ water and PBS 1×, respectively, were transferred into four wells each. Afterwards, 100 µL of erythrocytes suspension were added to each well containing test samples and controls. The plates were incubated for 1 h at 37 °C. Then, the plates were centrifuged for 10 min at 1000× *g* and 50 µL of the supernatant from each well was carefully collected and transferred for new microplates. The absorbance was measured at 570 nm with a reference filter at 630 nm using a BioTek ELx800 absorbance microplate reader (BioTek Instruments). The percentage of hemolysis was calculated for each sample in accordance with Equation (3):

(3) $$\mathrm{Hemolysis}\;\left(\%\right)=\frac{{\mathrm{OD}}_{S}-{\mathrm{OD}}_{N}}{{\mathrm{OD}}_{P}-{\mathrm{OD}}_{N}}\times 100$$

where OD_S_ is the optical density of the sample, OD_N_ is the average optical density of the negative control and OD_P_ is the average optical density of the positive control.

##### Preliminary In Vivo Safety Assays Using *Artemia salina*

The safety of AuNPs was assessed using *Artemia salina* (also known as brine shrimp) as in vivo testing model [66]. *Artemia salina* lethality assay has been widely applied to study the toxicity of heavy metals, ion metals and even nanoparticles, and it is mostly recognized as a cheap, fast, reproducible, easy and low-requirement method [66,67]. Artificial sea water was primarily prepared by dissolving commercial sea water salt in tap water, following the product instructions. Then, *Artemia salina* eggs were hatched within 48 h in artificial sea water by adding dry cysts in a recipient filled with artificial seawater and kept at a temperature from 25 to 30 °C, under aeration and continuous illumination. On the day that the nauplii were incubated with the particles, the aeration was interrupted before transferring 900 µL of artificial sea water containing 10–15 nauplii to each well from a 24-well plate, to allow the dead *artemia* and eggs either to sediment in the bottom of the growing container, or to float on the artificial sea water–air interface. Subsequentially, 100 µL of each testing solution was added to the wells containing the nauplii: AuNPs suspension from all Ratios (1280 ± 210 µM of Au in artificial sea water); artificial sea water only (as negative control); and 100% of DMSO (as positive control). The nauplii were exposed to testing solutions for 24 h, being kept in the same conditions of growth, but without aeration, and the number of dead nauplii was counted with the help of a magnifying glass. To kill the remaining live *artemia* 100 µL of 100% of DMSO was added. About 2 h later, the total *artemia* were counted and the mortality (%) was calculated according with Equation (4). As a note, many studies reporting brine shrimp lethality assays count the alive *artemia* [66,67,68], however, herein it was chosen to count the total number of dead nauplii to surpass the difficulties of having the exact same number of nauplii in all wells [66]. The mortality (%) of all AuNPs suspensions was calculated according to Equation (4), as follows

(4) $$\mathrm{Mortality}\;\left(\%\right)=\frac{{\mathrm{Dead}}_{24h}}{{\mathrm{Dead}}_{\mathrm{Total}}}\times 100$$

where Dead_24h_ is the number of dead nauplii after 24 h incubation with the samples and Dead_Total_ is the total number of nauplii in each well. All samples were tested twice with four replicates each time.

#### 2.2.7. Statistical Analysis

All results are represented as mean ± standard deviation (SD), for a specified n. All statistical analyses were carried out using GraphPad Prism 8^®^ (San Diego, CA, USA) and differences were considered significant when *p*-value < 0.05. One-way ANOVA followed by Tukey’s multiple comparisons test was used to compare the AuNPs’ features, and cell viability (%) and mortality (%) results upon incubation with the different AuNPs Ratios. To compare the AuNPs’ mean size determined by DLS and by AFM, one-way ANOVA followed by Sidak’s multiple comparisons test was used. 

## 3. Results

### 3.1. Physicochemical Characterization of AuNPs

#### 3.1.1. Size, PdI, Surface Charge and Maximum Absorbance Peak

**Figure 2:**
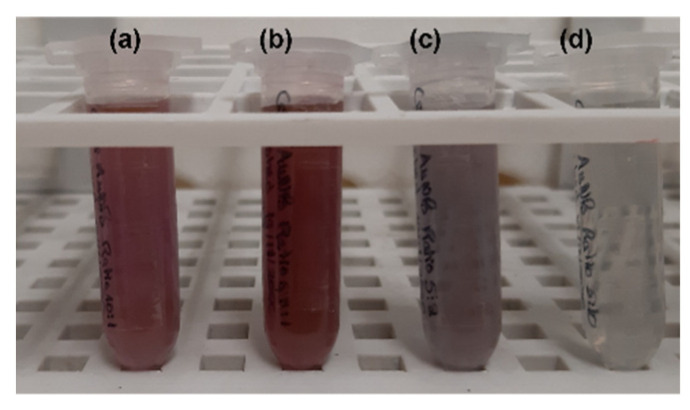
Macroscopic aspect of different suspensions of AuNPs. (**a**) Ratio 1; (**b**) Ratio 2; (**c**) Ratio 3; (**d**) Ratio 4.

The physicochemical characterization of the obtained AuNPs depending on the RA concentration used on their syntheses was carried out by DLS, electrophoretic mobility and spectroscopy techniques. A preliminary assessment of the success of the syntheses was carried out by analyzing the color of the colloidal suspensions obtained (Figure 2). The syntheses were considered successful when the gold salt color, initially yellow, changed upon reaction with the mixture of reducing agents, as shown in Figure 2. 

AuNP characterization data are gathered in Table 2. Considering the hydrodynamic diameter, the RA concentration used for AuNP syntheses did not significantly affect particle size, particularly for the lower Ratios. Nevertheless, for the maximum RA concentration, the resulting AuNPs were fairly larger, presenting average hydrodynamic diameters around 570 nm. In terms of PdI, the increase in the RA concentration resulted in AuNPs with higher PdI values (PdI > 0.3). This relation was more notorious and statistically significant for the two higher RA concentrations tested, with PdI values superior to 0.3 and 0.4 for Ratios 3 and 4, respectively. Moreover, by increasing the RA concentration, AuNP absorbance spectra were affected, and two different behaviors were noticed. AuNPs resulting from the use of lower RA concentrations (≤1.6 mM, Ratios 1 and 2) presented narrower, prominent, centered and almost symmetric peaks ranging from 549 to 561 nm. In contrast, AuNPs prepared with higher RA concentrations (Ratios 3 and 4) presented rather larger and broader absorbance bands with enhanced absorbance at longer wavelengths (≥600 nm). Lastly, significant differences in AuNP surface charge were observed between the formulations; however, it was not possible to establish a relationship between those differences and the RA concentration used.

#### 3.1.2. Quantification of the Synthesized AuNPs

Recovery yields calculated based on the Au content of each ratio are presented in Table 3. Differences were observed between tested Ratios, nevertheless they did not show a direct relationship with the RA concentration used for the AuNP syntheses. Ratio 2 showed the highest Au recovery yield among all formulations.

### 3.2. Morphological Characterization

**Figure 3:**
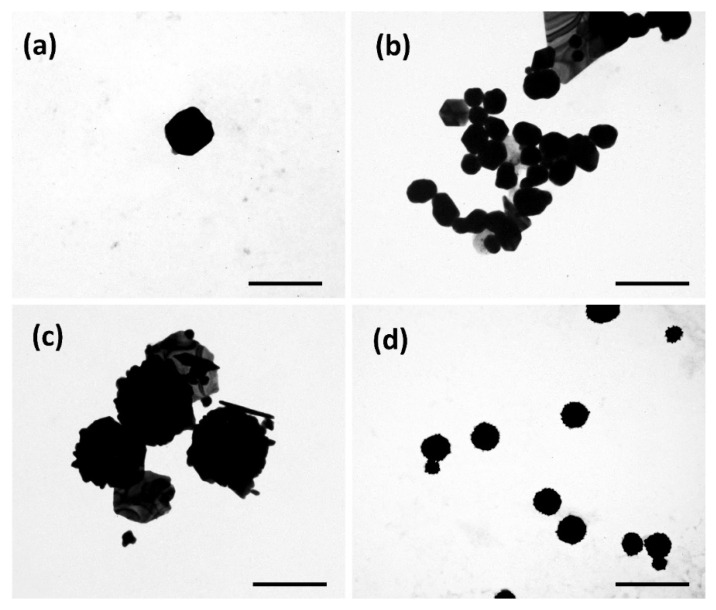
Morphological characterization of AuNPs by TEM. (**a**) Ratio 1, scale bar = 300 nm; (**b**) Ratio 2, scale bar = 200 nm; (**c**) Ratio 3, scale bar = 400 nm; (**d**) Ratio 4, scale bar = 400 nm.

**Figure 4:**
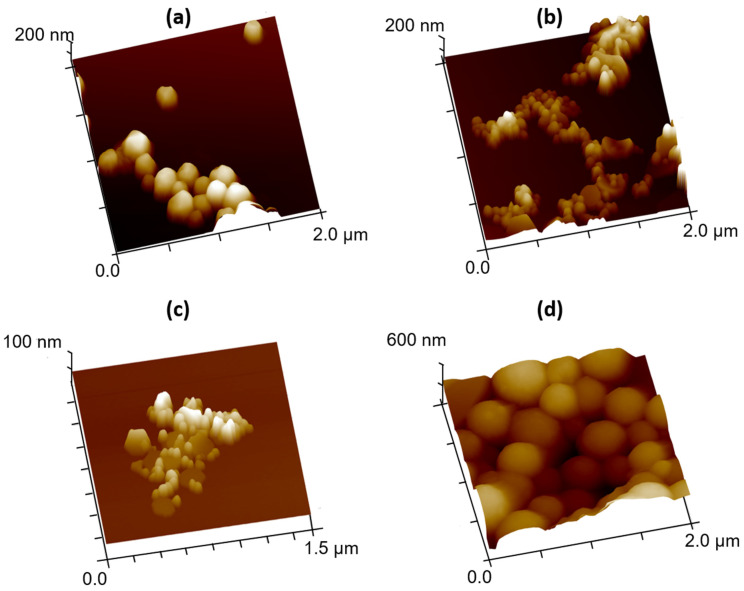
3D AFM images of AuNPs. (**a**) Ratio 1; (**b**) Ratio 2; (**c**) Ratio 3; (**d**) Ratio 4.

The effect of the RA concentration on the synthesis protocols over the AuNP morphology was assessed by TEM (Figure 3) and AFM (Figure 4). The images obtained by TEM and AFM showed that although most particles presented spherical or quasi-spherical shape, other planar structures were also observed, especially in the case of Ratio 2 and 3. The planar structures were herein denominated this way, because they exhibited basal dimensions, ranging from 100 nm up to 1 µm, which was much larger than their almost neglectable thickness. Moreover, TEM images of AuNPs from Ratios 3 and 4 showed some surface roughness in contrast to what was observed for the other Ratios tested.

**Figure 5:**
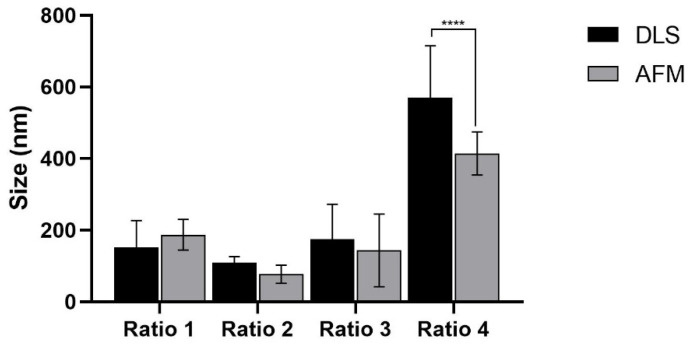
Comparative analysis of the AuNPs mean size determined by DLS (black columns) and AFM (grey columns). Data are represented as mean value ± SD, n ≥ 22. Statistical significance: **** *p* < 0.0001 comparing to the average hydrodynamic diameter of same type of particles determined by DLS.

Considering the limited accuracy of the DLS analysis for more polydisperse samples, particle sizes were also analyzed by AFM, and the comparative results are shown in Figure 5. No significant differences were observed between the two methodologies except for the case of AuNPs from Ratio 4. In this case, smaller sizes by AFM than by DLS were observed.

### 3.3. In Vitro Thermal Activation Studies Using Phantoms

**Figure 6:**
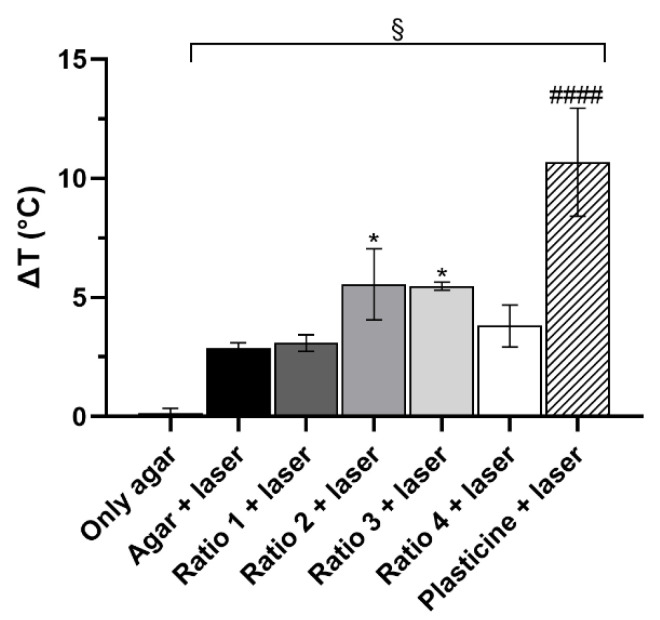
Phantom difference of temperature upon irradiation with a NIR laser for 3 min. The Au concentration in all 4 Ratios was the same as for the in vitro studies. The results represent the mean value ± SD, n = 4. Statistical significance represents: § *p* < 0.05 compared with the phantoms of only agar; * *p* < 0.05 comparing with the phantoms of only agar irradiated with the NIR laser; #### *p* < 0.0001 comparing with all the other phantoms.

AuNP thermal activation by NIR radiation was assessed upon their incorporation into agar phantoms and registering the temperature of the phantoms before and after laser irradiation. The differences in temperature measured for all the phantoms are shown in Figure 6. Phantoms of only agar were used as controls for possible temperature fluctuations caused by the laboratory temperature, and no temperature variations were noticed. These phantoms were posteriorly irradiated and used as controls of the temperature increment caused by the laser irradiation alone (due to direct heating of the thermocouple material), having been noticed an increase of about 3 °C. In turn, phantoms with a sphere of black plasticine were used as positive controls for temperature rise, since black plasticine strongly absorbs radiation and leads to high temperature increase. Phantoms incorporating a plasticine sphere presented a temperature increase of about 10 °C. The incorporation of Ratio 1 AuNPs did not seem to result in an increase in the phantoms’ temperature upon irradiation when compared to the phantoms of agar only also irradiated. The same was observed for Ratio 4 AuNPs. In contrast, Ratio 2 and Ratio 3 AuNPs resulted in increased phantom temperature of about 5.5 °C.

**Figure 7:**
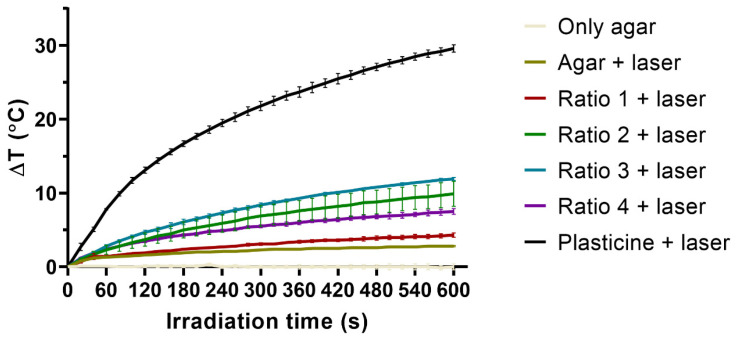
Evolution of the phantoms’ temperature as function of the irradiation time with a NIR laser. The Au concentration in all 4 Ratios was the same as for the in vitro studies. The results represent the mean value ± SD, n = 3.

The evolution of the phantoms’ temperature as function of the irradiation time was also assessed up to 10 min of irradiation, and data are depicted in Figure 7. In similarity to what was seen after 3 min of irradiation, no temperature fluctuations were observed for phantoms of only agar, and phantoms with plasticine showed the highest temperature rise. Moreover, phantoms of agar irradiated presented similar results to phantoms incorporating Ratio 1 AuNPs irradiated. In addition, as seen in the previous experiment, phantoms incorporating Ratio 2 AuNPs presented high variability of temperature that ranged from the highest temperatures reached by phantoms incorporating Ratio 3 AuNPs, to the lowest temperatures observed by those incorporating Ratio 4 AuNPs.

### 3.4. In Vitro Nanoparticle Internalization Studies Using Light Microscopy

**Figure 8:**
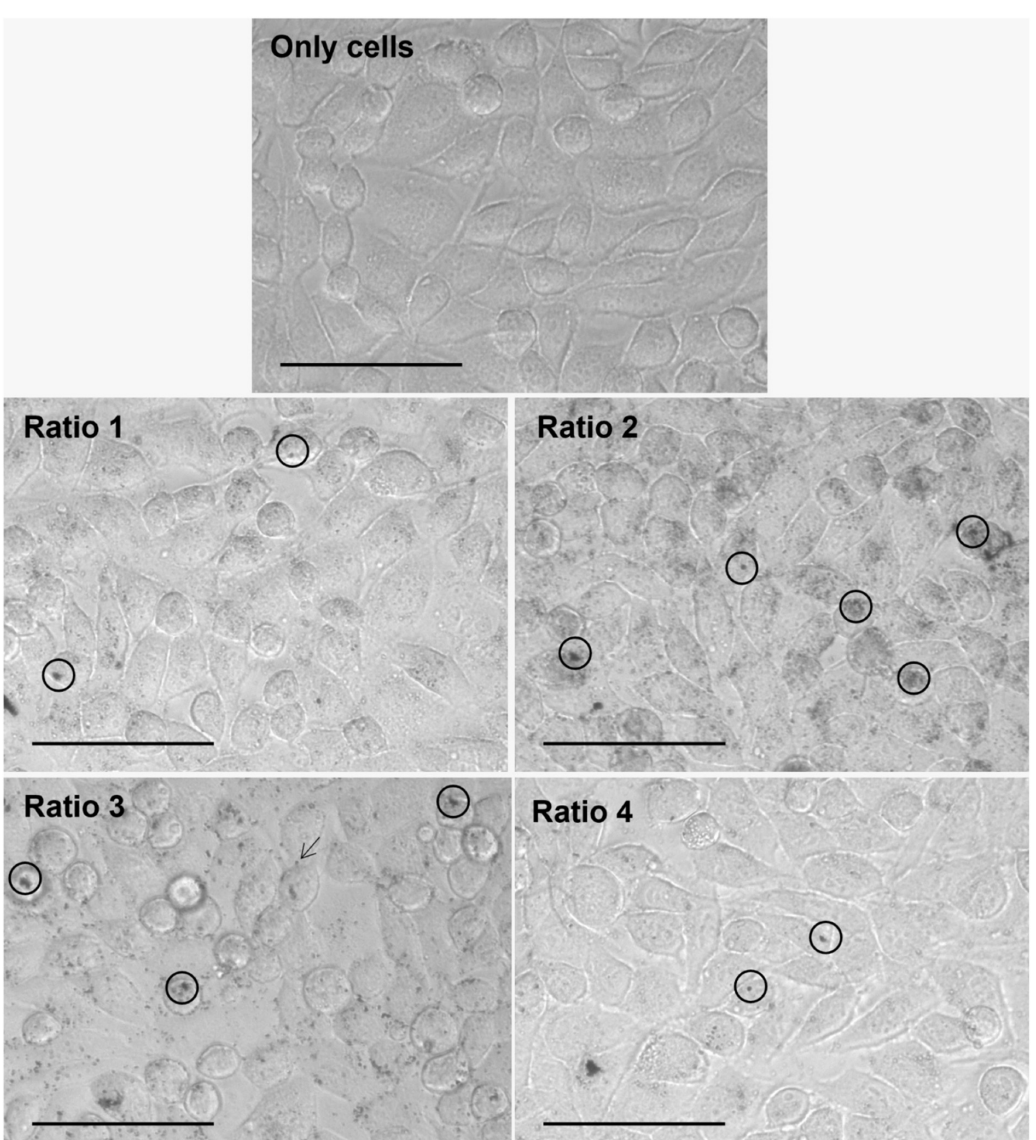
Representative images of L929 cells alone (Only cells) and after 4 h of incubation with different types of AuNPs (Ratio 1, Ratio 2, Ratio 3 and Ratio 4 AuNPs) at 40× magnification. AuNPs appear as darker structures either inside the cell (circle) or at the cell surface (arrow). Scale bar = 75 µm.

**Figure 9:**
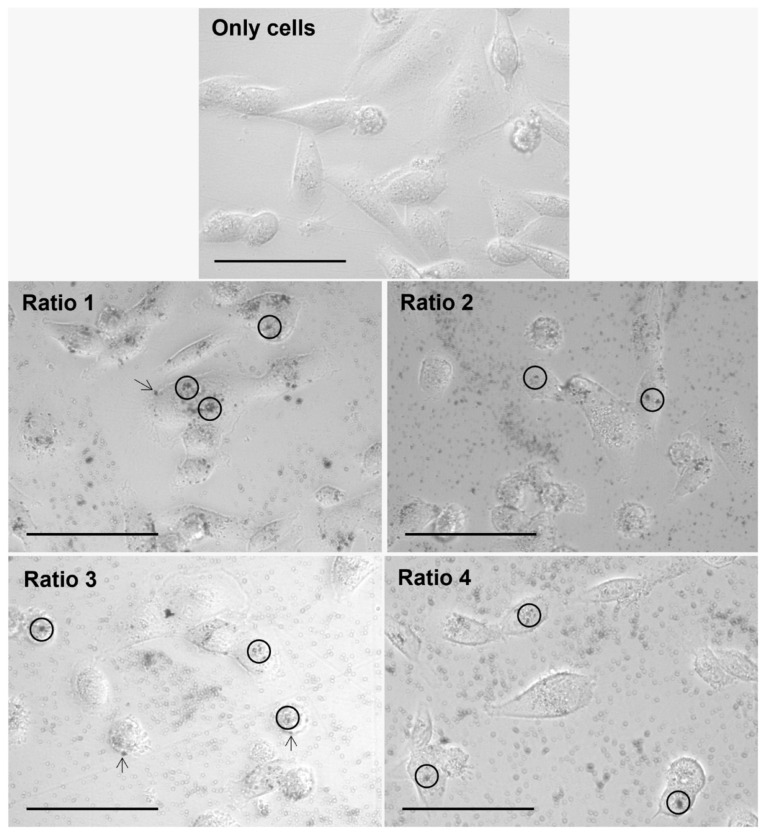
Representative images of MDA-MB-231 cells alone (Only cells) and after 4 h of incubation with different types of AuNPs (Ratio 1, Ratio 2, Ratio 3 and Ratio 4 AuNPs) at 40× magnification. AuNPs appear as darker structures either inside the cell (circle) or at the cell surface (arrow). Scale bar = 75 µm.

**Figure 10:**
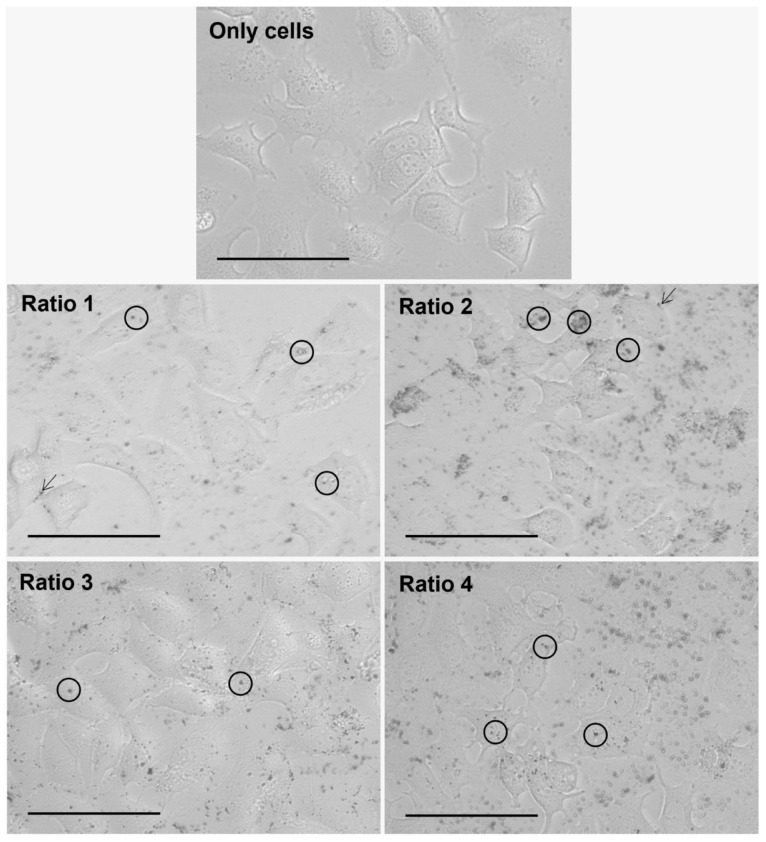
Representative images of MCF-7 cells alone (only cells) and after 4 h of incubation with different types of AuNPs (Ratio 1, Ratio 2, Ratio 3 and Ratio 4 AuNPs) at 40× magnification. AuNPs appear as darker structures either inside the cell (circle) or at the cell surface (arrow). Scale bar = 75 µm.

**Figure 11:**
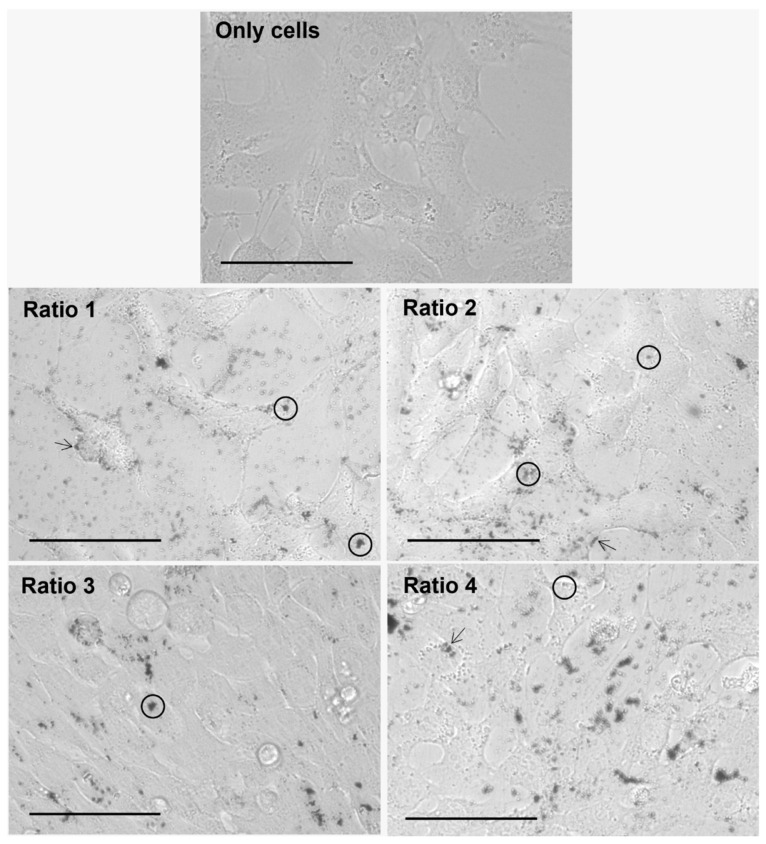
Representative images of 4T1 cells alone (only cells) and after 4 h of incubation with different types of AuNPs (Ratio 1, Ratio 2, Ratio 3 and Ratio 4 AuNPs) at 40× magnification. AuNPs appear as darker structures either inside the cell (circle) or at the cell surface (arrow). Scale bar = 75 µm.

The internalization of the different types of AuNPs after an incubation period of 4 h with L929, MDA-MD-231, MCF-7 and 4T1 cells was assessed by brightfield optical microscopy, and representative images are gathered in Figure 8, Figure 9, Figure 10 and Figure 11, respectively. This technique represented a first approach to assess the internalization of the AuNPs, based on how the focus of the presumed AuNPs changed when changing the focus of the cells. It was assumed that particles changing simultaneously with the focus of the cells were internalized, whereas particles whose focus was not affected by the focus of the cells were considered adsorbed to the cell membrane.

Furthermore, considering the results observed for all cell lines and AuNP Ratios under test, a table scoring the AuNPs internalization for each cell line was created (Table 4). As a note, in the case of 4T1 cells, it was very difficult to evaluate the presence of AuNPs once the cells present naturally appearing black “spots”. Moreover, additional difficulties were encountered when delimiting cells, and thus evaluating whether the particles were internalized, adsorbed or simply in the medium.

### 3.5. Safety of AuNPs without Laser Irradiation

**Figure 12:**
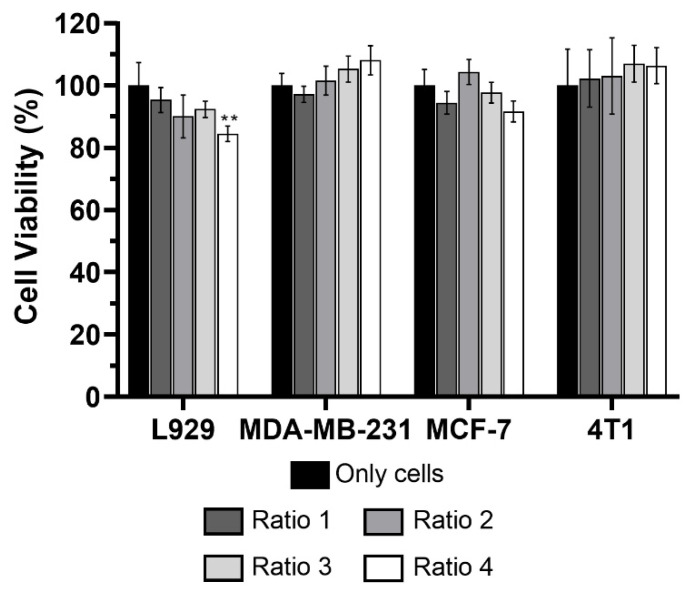
Cell viability (%) of L929, MDA-MB-231, MCF-7 and 4T1 cells incubated for 24 h in complete medium (black columns) and with different types of AuNPs (Ratio 1, Ratio 2, Ratio 3 and Ratio 4 AuNPs) at a concentration of 128 ± 21 µM in terms of Au content. The results represent the mean value ± SD, n ≥ 5. Statistical significance represents: ** *p* < 0.01 compared with the cells from the same line incubated in complete medium, which represent 100% viability.

The safety of the different types of AuNPs produced was initially assessed in vitro in L929, MDA-MB-231, MCF-7 and 4T1 cells incubated for 24 h with 128 ± 21 µM in terms of Au content, and viability was assessed using MTT assay (Figure 12). None of the AuNPs seemed to affect cell viability, except for Ratio 4 AuNPs which led to a slight reduction in L929 cell viability.

**Figure 13:**
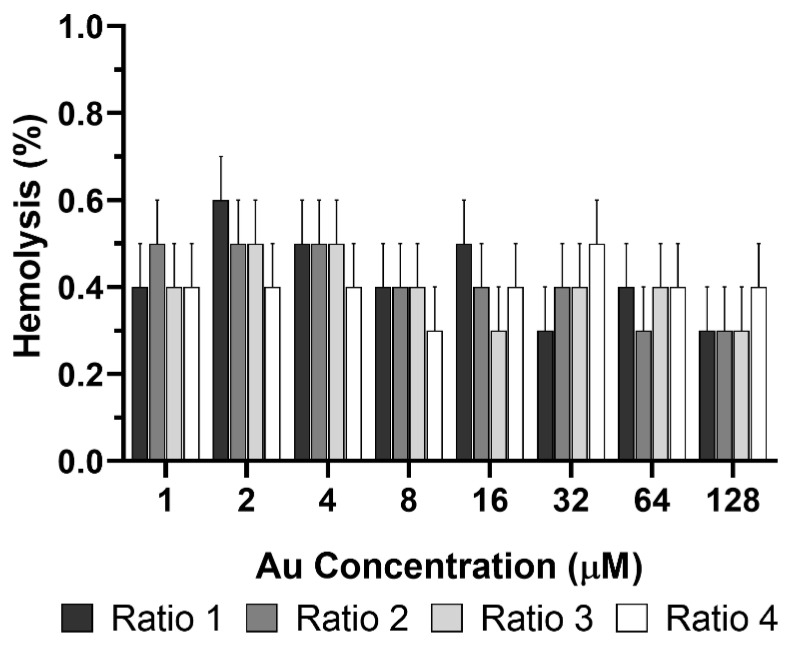
Hemolytic activity of different types of AuNPs (Ratio 1, Ratio 2, Ratio 3 and Ratio 4 AuNPs). PBS 1× (pH 7.4, USP32) and MilliQ water were used as negative and positive controls, respectively. Results represent the mean value ± SD, n ≥ 3.

The safety of the AuNPs with other human cells was also performed. In this case, red blood cells were used and the hemolytic activity of the different AuNPs was determined using EDTA-preserved peripheral human blood. Results showed no hemolytic effect for all tested AuNPs Ratios (Figure 13). The maximum concentration tested was the same as the one used in in vitro assays depicted in Figure 12, while the minimum corresponded to 1 µM in terms of Au content. These results indicate that even if the AuNPs, aimed to be administered in situ, reach the blood stream, they would not cause erythrocyte lysis, and thus should be safe to be used in vivo.

**Figure 14:**
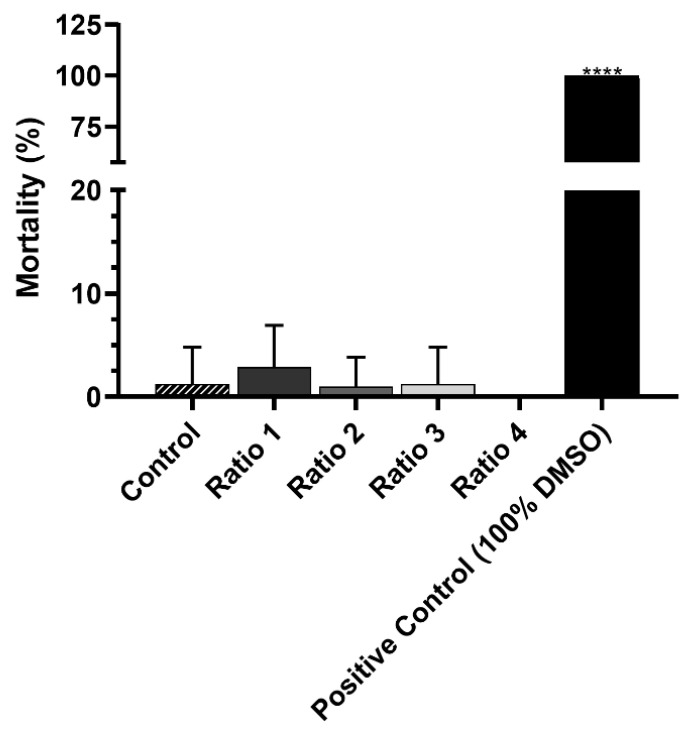
Mortality (%) of *Artemia salina* incubated for 24 h with only artemia salt medium (Control), with different types of AuNPs (Ratio 1, Ratio 2, Ratio 3 and Ratio 4 AuNPs) at Au concentration of 128 ± 21 µM, and with 100% of DMSO as positive control of mortality (%). The results represent the mean value ± SD, n = 8. Statistical significance represents: **** *p* < 0.0001 compared with the group incubated only with artemia salt medium.

Lastly, the safety of the AuNPs was preliminarily assessed in *Artemia salina*, a simple in vivo model commonly used for toxicity evaluation. As shown in Figure 14, none of the formulations presented in vivo toxicity, in contrast with the positive control (100% of DMSO), therefore confirming the safety of the synthesized AuNPs.

## 4. Discussion

This work describes the synthesis of AuNPs using an alternative chemical method based on the use of a mixture of reducing reagents including a natural-like reagent, RA, in substitution of toxic reducing agents, such as CTAB. Furthermore, the RA concentration was varied and the resulting AuNPs were thoroughly characterized in terms of physicochemical and morphological features, as well as in terms of in vitro and in vivo safety. Results showed that the RA concentration used in the AuNPs syntheses presented an active role over the AuNPs features, allowing the tunability of certain features for later use of these AuNPs as part of a PTT system. 

AuNPs can be produced via chemical, electrochemical and physical approaches [43,47,48], with chemical approaches being the oldest and more commonly used [42,43,44]. Typically, chemical approaches use reducing agents to reduce the metal salt [43,44,48], and sometimes they may also include the use of capping agents [41]. The most frequent synthetic methods require the use of harmful reagents both for the environment and biological entities [43,44,45]. Nevertheless, alternative approaches were already proposed using environmentally friendly and minimally toxic reagents such as plant extracts, algae, fungi, and bacteria, among others [47,49,69]. Among the biological sources used as reducing and capping agents for AuNPs syntheses, *Plectranthus saccatus* Benth plant extract has previously been used by our group to obtain a mixture of spherical and rod-shaped AuNPs with an average size around 200 nm and maximum absorbance wavelength within the NIR range [52]. This plant extract was, however, hard to obtain and resulted in high variability of the AuNPs shape, which encouraged its substitution by RA, its main constituent [52,70]. The use of RA in the synthesis of AuNPs has been previously proposed by others, nevertheless the synthesis methods were different and the particles final application was unknown or different than the one proposed here [71,72]. In 2010, Sarkar and colleagues used RA in combination with caffeic and dihydrocaffeic acids as reducing agents on the synthesis of AuNPs. The group proved the production of AuNPs with sizes around 20 nm; however, no further characterization was reported, nor was a final application of the AuNPs proposed [71]. In 2018, Lim et al. used RA as reducing and stabilizing agent in the synthesis of AuNPs, preparing negatively charged particles with sizes around 30 nm, mostly spherical but also with planar shapes (triangles, hexagons and pentagons) [72]. Their objective was to characterize particles’ physicochemical properties and to evaluate their catalytic activity for possible biotechnological, chemical, and industrial applications. They also assessed the absorbance spectra of the AuNPs, which showed a maximum absorbance peak at 532 nm, a wavelength within the visible range and thus not optimal for PTT applications. Therefore, all methods using RA previously reported for the synthesis of AuNPs resulted in small AuNPs (around 20–30 nm) with unknown absorbance or absorbance within the visible range of the light. These features do not fulfil the basic requirements predefined by our group for a future in situ application of the AuNPs as part of a PTT system for treatment of breast cancer, because they are too small and do not absorb light preferentially at wavelengths within the NIR region. Moreover, to the best of our knowledge, there are no studies on the possible effect of the RA concentration used in the AuNP synthesis on AuNP physicochemical and biological effects. 

A preliminary assessment on the success of the syntheses relied on the analysis of the color of the colloidal suspensions. Before reacting, the gold salt solution presented a yellow color, which then changed after reaction with the mix of reducing agents. The color of the syntheses varied according to RA concentration used, which led us to hypothesize that the RA concentration indeed played an active role on the features of the AuNPs obtained. This hypothesis was firstly considered because it is well-known that the color of colloidal gold is related to the physicochemical and morphological properties of the AuNPs [73]. Apart from proving the active role of RA on the AuNP syntheses, the visual analysis of the colloidal suspension color and aspect also gave an idea on the solution concentration. Ratio 2 synthesis is more concentrated than the remaining solutions since its color is far stronger and less transparent. Contrarily, Ratio 4 synthesis appears very transparent, which questions its concentration. These suspicions agreed with absorbance values later detected by spectrophotometry, with Ratio 2 AuNPs showing the strongest absorbance values and Ratio 4 AuNPs the lowest absorbance values (about 4 times inferior to the remaining samples). Then, to obtain information on the colloidal suspension concentrations and on the reaction yield, samples were analyzed by ICP-MS to quantify the Au content. Ratio 2 presented the highest Au content, which agreed with the color density observed both by macroscopical and spectroscopic analysis. Moreover, no significant differences were observed between the other Ratios, which was not expected for Ratio 4 AuNPs since it seemed to be the least concentrated sample. The exact reason behind these results was not completely understood; however, it is hypothesized that this might occur because Ratio 4 solutions present larger particles in fewer amounts, compensating the reduced number of particles with its size. Another important aspect considering attending the recovery yield based on the Au content, is that a high portion of the Au used in the synthesis is not reacting (>38%). This means that there is still plenty of room for improving the synthetic method either by decreasing the Au salt concentration used in the syntheses, by increasing the proportion of reducing agents or by adding other reducing agents with stronger reducing power.

The size of the AuNPs obtained seemed not to be affected by the RA concentration in the case of the smaller concentrations tested (Ratio 1, 2 and 3), leading to the production of particles with sizes ranging between 110 and 175 nm. As a note, the sizes herein mentioned are referred to DLS analysis and agree with the values observed by AFM analysis upon averaging the diameter of random AuNPs. In contrast, AuNPs produced by using the highest RA concentration (Ratio 4) were fairly larger, presenting an average size of 570 nm according to DLS analysis. When considering the sizes obtained by AFM analysis, Ratio 4 AuNPs were slightly smaller, showing average sizes of 414 ± 60 nm. This difference in size between the two techniques was previously reported by others [74,75], and the high polydispersity index of the particles suspensions were pointed out as one of the main reasons behind it. DLS is a fast, inexpensive, simple and easily accessible technique, thus being considered a primarily suitable approach to determine the particle size distribution [76,77]. Nevertheless, it has some drawbacks, especially for polydisperse samples, lacking the ability to distinguish different particle populations with similar sizes, and small aggregates from larger particles [78]. Moreover, according to ISO 22412, 2017, the size of the particles measured by DLS can be biased for bigger values when the samples are strongly polydisperse (PdI > 0.7) [79,80]. In turn, AFM is a microscopic technique that provides information on particle morphology and surface roughness, as well as on their size [75]. For size analysis, AFM was reported to be more advantageous than DLS for the identification of different populations of AuNPs within polydisperse AuNP suspensions [74,75]. Another factor that is also hypothesized to play a role on the differences between the two techniques is the form of the samples [75]. Typically, DLS analysis is performed in wet suspensions of particles (e.g., aqueous suspensions), whereas AFM analysis is performed in dried samples after evaporation of the suspension solvent when deposited onto a rigid substrate, which raises the hypothesis that the immobilization method selected to prepare the AFM sample might affect the results [75]. Both DLS and AFM present advantages and disadvantages, and the choice of which one to use depends not only on the availability of the equipment, but also on the type and polydispersity of the samples. Thus, the combination of both techniques might be advantageous for the overall characterization. The ideal size of AuNPs for biomedical applications is still debatable since it will depend on the specific application and targeted cells/tissues [41,43,81,82,83]. Moreover, in addition to the biological effect, the size of the particles will also influence the AuNP optical and physicochemical properties [41]. Herein, the goal was to develop AuNPs with suitable properties to be used as photothermal enhancers after being administered in situ for PTT purposes. Therefore, AuNPs must present sizes that confer them the ability to be retained at the tumor site (injection site) with minimal-to-absent migration to non-targeted organs, while simultaneously delaying their removal by elements from the immune system. In general, smaller AuNPs present a far wider biodistribution than larger particles (>200 nm) [43,48,50,84], and AuNPs ability to cross both the blood–brain and placenta barriers when sizes are smaller than 20 nm was already reported [85,86,87]. Moreover, AuNPs larger than 200 nm can hardly cross the haemato–intestinal barrier or the skin, when compared to smaller AuNPs [85,88]. Considering the aforementioned information and the final purpose of the synthesized AuNPs, particles with sizes around 200 nm seem beneficial to enhance local particle accumulation at the tumor site and hinder migration to other non-targeted organs [51]. Thus, when considering only the size factor, Ratio 3 AuNPs seem to be the most promising particles for further studies.

The polydispersity of the AuNPs synthesized proved to be affected by the RA concentration. The use of increased concentrations of RA resulted in more polydisperse populations of particles, with Ratio 1 and 2 AuNPs presenting an average PdI < 0.3, whereas Ratio 4 AuNPs presented PdI > 0.45. The PdI reflects the homogeneity of the particle population, and according with the ISO 22412:2017, 2017, a PdI < 0.07 is associated with a monodisperse sample of spherical particles. Moreover, samples with a PdI < 0.4 are considered homogeneous, >0.4 less homogeneous, and ≈1 completely heterogeneous [89]. The election of a threshold PdI value is still not consensual, with some stating values <0.3 as optimum [80], others considering values ≤0.5 still acceptable [90], and others setting the threshold as ≤0.7 [91]. In the present work, considering the PdI, both Ratios 1 and 2 are better than the remaining.

Another relevant parameter to consider is the AuNP surface charge, which will influence stability, solubility, toxicity, permeability, and in vivo circulation time [41,48,92]. Thus, to anticipate and optimize the particles’ biological effect, it is necessary to determine the AuNPs’ charge. Typically, neutral particles suffer less opsonization than charged particles [93]. Moreover, among charged particles, positively charged ones are more toxic and less stable than their negative counterparts [94,95], which encourages the use of negatively-charged AuNPs rather than positively charged ones. This election is also supported by some in vivo results previously reported, showing enhanced accumulation of negatively charged particles in tumors [96]. So, for this work, neutral-to-negatively charged particles were preferred, even though no reference value was established. Upon analyzing the surface charge of the AuNPs synthesized, all the AuNPs presented negative charge regardless of the RA concentration used. Some variations on the charge of AuNPs from different Ratios were noticeable, yet no relationship between the surface charge and the RA concentration could be established.

The morphology of the AuNPs is another important aspect to assess since it will influence not only their optical properties but also biological interactions and effects [37,41]. The AuNPs here obtained showed mostly spherical or quasi-spherical shape, independently of their ratio. Planar structures were also observed both by TEM and AFM, which supported the PdI values determined by DLS. This agrees with the results reported by Lim and coworkers when also using RA as reducing agents in the synthesis of AuNPs [72]. Moreover, a certain degree of heterogeneity is also associated with green-like synthetic approaches, showing the formation of particles with different shapes [97].

Keeping in mind the final application of the synthesized AuNPs, the absorbance spectra is a crucial comparison factor between different types of particles. As mentioned before, the main purpose of this work was to optimize the AuNPs’ synthesis and thoroughly characterize the resulting AuNPs with the intent of later using these particles for PTT. Hence, absorption of the light at wavelengths near the wavelength of the light source used in the PTT system is key to enhance the conversion of the light energy into heat [98]. In our group, a NIR laser is used for the proposed PTT strategy [54,55,56] to potentiate a deeper penetration of light into tissues. Therefore, AuNPs showing maximum absorbance peak wavelengths within the NIR region (650–900 nm) are preferable. In addition, AuNPs showing broader absorbance bands are also of interest, allowing particle versatility to be combined with lasers with different wavelengths [99,100]. The synthesized AuNPs presented two different patterns of absorbance spectra depending on the RA concentration used on the syntheses. AuNPs obtained using lower concentrations of RA (Ratio 1 and 2) showed maximum absorbance peaks centered around 555 nm, which belongs to the visible range. In turn, AuNPs synthesized with higher concentrations of RA (Ratio 3 and 4) presented broader absorbance peaks with enhanced absorbance within the NIR range. Ratio 3 and 4 AuNPs showed preferable features for a future application in a PTT system. Nevertheless, Ratio 4 AuNPs had an impactful drawback in this regard. The absorbance intensity of Ratio 4 AuNPs was about 4 times smaller than the one of Ratio 3 AuNPs, which raises doubts over the photothermal properties of those particles. Therefore, Ratio 3 AuNPs seemed by far the most promising AuNPs in terms of photothermal properties. The photothermal properties were preliminarily assessed by studying the thermal activation of the AuNPs once incorporated into agar phantoms and upon their irradiation with a NIR laser (811 nm wavelength) up to 10 min. All phantoms showed similar trace trends: an initial quick temperature rise followed by an approach to a plateau-like state, which agrees with works already reported [101,102]. Results showed that Ratio 1 and Ratio 4 AuNPs were unable to cause temperature rises significantly higher than the ones caused by simple irradiation of agar phantoms without particles, showing their poor photothermal properties. These results were partially expected, since Ratio 1 presented preferable absorbance at 560 nm (far from the NIR range) and Ratio 4 seemed to be very poorly concentrated. In turn, Ratio 2 and Ratio 3 AuNPs led to a significant increase of the phantom temperature when comparing to the increase observed for the irradiated phantoms without particles. The enhanced photothermal properties of these AuNPs were very positive results and highlighted the potential of these two Ratios. The results for Ratio 3 AuNPs might be a consequence of the AuNP preferential absorbance at wavelengths near the NIR region. Nevertheless, AuNPs from Ratio 2 absorb preferentially at wavelengths around 550 nm, which is far from the NIR range. The reason behind the similar results observed for these two Ratios is not fully understood; however, it is hypothesized that they might be related to the amount of AuNPs present in each sample. Even though all the samples were tested at the same concentration of Au element, it is suspected that the number of the particles in the suspension is different. This hypothesis was raised because Ratio 2 AuNPs suspensions showed a stronger color and opacity than Ratio 3 AuNPs, as well as a higher absorbance signal, even after uniformizing the Au concentration in all the formulations tested.

The AuNP ability to be internalized by both human and murine cells was assessed by brightfield optical microscopy upon an incubation period of 4 h. The incubation period was chosen based on data previously obtained from our group [53,54]. Optical microscopy analysis showed the successful internalization of the particles in a diversity of murine fibroblast and human and murine breast cancer cells. Complementary methodologies such as fluorescence or confocal microscopies could be tested in the very near future. Nevertheless, our results indicate that AuNP internalization and/or surface adsorption depends on the tested cell line. Furthermore, Ratio 2 and 3 AuNPs seem to be preferentially internalized or adsorbed in comparison with the AuNPs from other Ratios. 

After assessing the internalization of the AuNPs, their safety was evaluated in the same cells using the MTT assay. None of the AuNPs tested significantly reduced cellular viability upon an incubation period of 24 h. The exception was Ratio 4 AuNPs, which reduced cell viability of fibroblasts in 15%. Despite this reduction, the AuNPs are still considered safe for all the cell lines at the concentration tested based on the cytotoxicity definition in ISO 10993-5:2009 (E) stating that a formulation or product is considered cytotoxic when the cellular viability is reduced in more than 30%. The safety of AuNPs was also proved in human red blood cells. For the intended application of the AuNPs, local administration will minimize the risk of reaching the blood stream. Nevertheless, it is important to ensure that even if the administered AuNPs get to the circulatory system they will not cause hemolysis. At last, the safety of the AuNPs was assessed in *Artemia salina*, a preliminary in vivo model commonly used for toxicological assays [66,67]. Results confirmed the safety of AuNPs at the concentration tested and for this in vivo model.

In summary, the method herein reported allows the preparation of AuNPs with variable physicochemical and optical properties, depending on the concentration of the RA solution used. Among the AuNPs and considering a future application of these AuNPs for PTT approaches in breast cancer, Ratio 3 AuNPs appear as the most promising particles, with not only a mean size that might potentiate their local accumulation at the tumor site, but also a broader absorbance peak with enhanced absorption close to the NIR region, presumably with enhanced photothermal effect. Moreover, the therapeutic value of these AuNPs can still be improved by adding, for instance, specific coatings and/or targeting moieties to enhance not only the photothermal effect but also the AuNPs selectivity towards tumor cells.

## 5. Conclusions

AuNPs with different features were successfully prepared after adapting a method that we have recently proposed. By changing the RA concentration used in the syntheses, AuNPs with a wide range of sizes (100–570 nm) and polydispersity index (0.2–0.5) were obtained. Moreover, AuNP surface charge seemed to be equally affected by RA concentration, although all the AuNPs presented negative charge no matter the RA concentration. In addition, RA concentration also affected the AuNPs absorbance spectra, with a shift of the AuNPs maximum absorbance peak towards the NIR region with increasing RA concentrations. By its turn, the obtained AuNPs also proved to be able to differentially increase the temperature of the surrounding media upon being irradiated with a NIR laser. Lastly, the AuNPs were safe in vitro in human and murine cell lines and in human red blood cells, as well as in a preliminary in vivo model using *Artemia salina*. Together, these results show the successful preparation and thorough characterization of synthesized AuNPs using a recently developed method proposed by the group, that proves the active role of RA on the nanoparticles’ features. Moreover, the results attained highlight the potential of using the produced AuNPs as part of a photothermal therapy system. Even though there is still room for improving the methodologies herein reported, the overall results are very promising and draw particular attention to the AuNPs of one of the Ratios (Ratio 3), which could in the future be coated and/or conjugated with targeting moieties to simultaneously improve the photothermal and targeting properties of the particles. Thus, the AuNPs prepared show high potential to be further modified and act as PTT enhancers in a PTT system combining AuNPs with a NIR laser for the treatment of breast cancer. 

## Figures and Tables

**Table 1:** Nomenclature used to identify AuNPs, RA concentration and overall mass percentage.

AuNPs Nomenclature	RA Concentration (mM)	RA Mass Percentage (*m*/*m*) *
Ratio 1	0.9	8%
Ratio 2	1.6	14%
Ratio 3	3.5	26%
Ratio 4	17.5	64%

* This establishes the relationship between the mass of RA and the total mass of reagents.

**Table 2:** AuNPs physicochemical characterization.

AuNPs	Maximum Absorbance Peak (nm)	Hydrodynamic Diameter (nm)	PdI	Zeta-Potential (mV)
Ratio 1	561 ± 17	151.1 ± 74.6	0.240 ± 0.071	−15.7 ± 5.4
Ratio 2	549 ± 14	108.8 ± 17.5	0.293 ± 0.077	−22.6 ± 2.1 **
Ratio 3	628 ± 88	175.0 ± 97.5	0.337 ± 0.085 ***	−21.8 ± 2.5 *
Ratio 4	830 ± 93 ****^,####,++++^	570.5 ± 144.2 ****^,####,++++^	0.462 ± 0.107 ****^,####,++++^	−15.9 ± 3.2 ^###,++^

For spectroscopy and DLS analyses, AuNPs were suspended in MilliQ water. For electrophoretic mobility analyses, AuNPs were suspended in PBS 1× (pH 7.4, USP32). Data are represented as mean ± SD, with n > 3. Statistical significance is represented as * *p* < 0.05, ** *p* < 0.01, *** *p* < 0.001 and **** *p* < 0.0001 comparing with Ratio 1 AuNPs; ^###^ *p* < 0.001 and ^####^ *p* < 0.0001 comparing with Ratio 2 AuNPs; ^++^ *p* < 0.01 and ^++++^ *p* < 0.0001 comparing with Ratio 3 AuNPs.

**Table 3:** AuNP recovery yield in terms of the Au element concentration.

AuNPs	Recovery Yield ^1^ (% (*n/n*))
Ratio 1	39 ± 8
Ratio 2	62 ± 13 *
Ratio 3	48 ± 10
Ratio 4	43 ± 9

^1^ Quantification by ICP-MS, considering only the Au element. Represented by mean ± measurement uncertainty, n = 2. Statistical significance represented as * *p* < 0.05 comparing with Ratio 1, Ratio 3, and Ratio 4 AuNPs.

**Table 4:** Score of AuNPs internalization per cell line by brightfield optical microscopy.

AuNPs	Ratio 1	Ratio 2	Ratio 3	Ratio 4
L929	++	++++	+++	+
MDA-MB-231	+++	++	++	+
MCF-7	+	+++	++	++
4T1	+	+	+	+

+ Poor presence of AuNPs in the cells (internalized or adsorbed). ++ Presence of AuNPs in the cells. +++ High presence of AuNPs in the cells. ++++ Abundant presence of AuNPs in the cells.
